# Contributors to nonsuicidal self-injury in incarcerated youth

**DOI:** 10.1186/s40352-017-0058-x

**Published:** 2017-12-13

**Authors:** Larkin Street McReynolds, Gail Wasserman, Elise Ozbardakci

**Affiliations:** Columbia University, New York, NY USA

## Abstract

**Background:**

Despite elevations in risks associated with self-injurious behavior among community adolescents, the degree to which these features are associated with self-injury among incarcerated youth has rarely been examined. Although the DSM-5 recently proposed a distinct category of nonsuicidal self-injury (NSSI), most studies of youths in forensic settings have not distinguished between subtypes of self-harming individuals.

**Methods:**

Demographic, offense, and disorder contributors to NSSI in incarcerated youths of both genders (N = 358) were examined via a computerized self-report instrument (VISA), largely consistent with DSM-5.

**Results:**

Nonsuicidal self-injurers (vs. non-injurers) were almost three times as likely to be white, slightly younger, and more than seven times as likely to have also made a suicide attempt. While males and females reported different rates of exposure to different types of assaultive violence, both nonsexual assault and forced sexual activity were approximately twice as likely among those reporting NSSI in both genders.

**Conclusion:**

Finding support standardized, universal screening for nonsuicidal self-injury in juvenile justice secure care facilities.

## Background

Elevated rates of self-injury have been noted in incarcerated populations (Chapman, Gratz, & Turner, 2014; Smith & Kaminski, 2011) including adolescents in secure care (Casiano, Katz, Globerman, & Sareen, 2013; Dixon-Gordon, 2011). A higher rate of self-injury in adolescent forensic samples is consistent with their increased prevalence of both psychiatric disorder (e.g., Wasserman, McReynolds, Schwalbe, Keating, & Jones, 2010) and trauma exposure (Wasserman & McReynolds, 2011).

Multiple terms are in use to describe self-injurious behavior, with variation in specificity. At the most general level, “self-injury” or “self-harm” refers to the act of deliberately harming one’s own body. The DSM-5 includes, as a “condition for further study”, a more specific subcategory of nonsuicidal self-injury (NSSI) with absence of suicidal intent. The alternative DSM-5 subcategory, suicidal self-injury (SSI), considers acts where the intent is to die. Investigations of adolescent self-harm have not always distinguished between the two subgroups of SSI and NSSI (e.g., Asgeirsdottir, Sigfusdottir, Gudjonsson, & Sigurdsson, 2011; Brunner, Parzer, Haffner, Steen, Roos, Klett & Resch, 2007; Ross & Heath, 2003). In adolescent community samples, rates of generally-defined “self-injury” are approximately 35%, while rates for the narrower constructs range between 14% and 22%, and 9% and 10% for NSSI and SSI, respectively (Jacobson & Gould, 2007).

Studies among adolescents and young adults are inconsistent in the degree to which they report gender differences in the rates of NSSI. Studies of community adolescents/young adults have sometimes reported higher rates of NSSI for females (Sornberger, Heath, Toste, & McLouth, 2012); a recent meta-analysis indicated higher prevalence among females (Bresin & Schoenleber, 2015). On the other hand, two meta-analyses have concluded that gender differences in NSSI appear less consistently among non-clinical populations (Swannell, Martin, Page, Hasking, & St.John, 2014; Van Camp, Desmet, & Verhaerghe, 2011). However, two investigations among incarcerated youth that included sufficient numbers of both genders to examine differences (Casiano et al., 2016; Ellis et al., 2002), higher rates of self-injury were found among females. Some evidence indicates a secular trend, such that the female preponderance among nonsuicidal self-injurers may be decreasing over time (Van Camp et al., 2011), but inconsistencies across investigations in their report of gender differences might also reflect whether or not they take other disorder conditions into account.

Risks linked to generally-defined self-injury in community samples of adolescents include internalizing conditions (e.g., Major Depressive Disorder, Post-traumatic Stress Disorder; Muehlenkamp & Gutierrez, 2007; Ross & Heath, 2002), externalizing behaviors (e.g., Conduct Disorder, Marijuana Abuse/Dependence; see Jacobson & Gould, 2007 for a review), and history of child maltreatment (Chapman et al., 2014; Fliege, Lee, Grimm, & Klapp, 2009; Gomez, Becker-Blease, & Freyd, 2015; Lang & Sharma-Patel, 2011). Several recent studies reported significant elevations in trauma exposure among adolescents who self-harm (Ellis et al., 2002; Kenny et al., 2008; McReynolds & Wasserman, 2011; Morgan & Hawton, 2004). Although theoretical delineations of self-injury etiology stress the contribution of a history of sexual abuse (van der Kolk, Perry, & Herman, 1991), there is disagreement regarding a unique connection between *sexual* abuse and self-injury (Gladstone, Parker, Mitchell, Malhi, & Austin, 2004; Klonsky & Moyer, 2008), since types violence exposure (i.e., sexual and non-sexual) often co-occur. Few studies of self-injury consider both physical and sexual abuse histories, each of which could have both direct and indirect (mediated by increased rates of mental health disorder) associations with self-injury. Two reviews of the sex abuse/self-injury association (Jacobson & Gould, 2007; Lang & Sharma-Patel, 2011) note added contributions of physical abuse. In a case-control study of adult ER admissions for self-injury, both sexual and physical abuse histories were elevated approximately three-fold among those reporting self-injurious behaviors (Newman & Bland, 2007). The clinical features associated with self-injury might be expected to vary depending upon presence/absence of co-occurring suicidal intent, although investigations of adolescents have not always distinguished between the two subgroups of SSI and NSSI, so that the differential clinical and trauma-related correlates of NSSI remain less clear.

Although many of these risks (e.g., trauma exposure, disorder) are elevated in adolescent forensic samples, the degree to which they are associated with NSSI in those populations has rarely been examined. In seven studies (Casiano et al., 2016; Ellis, Gormley, Ellis, & Sowers, 2002; Kenny, Lennings, & Munn, 2008; Matsumoto, Yamaguchi, Chiba, Asami, Iseki, & Hirayasu, 2005; McReynolds & Wasserman, 2011; Morgan & Hawton, 2004; Penn, Esposito, Schaeffer, Fritz, & Spirito, 2003) that compared the characteristics of incarcerated juveniles who have and have not self-injured themselves, most reported higher rates of both internalizing and externalizing concerns among those who self-harm (although not always significantly so). Four of these seven studies reported significant elevations in trauma exposure among self-harming adolescents (Ellis et al., 2002; Kenny et al. 2008, McReynolds & Wasserman, 2011; Morgan & Hawton, 2004). Some report higher rates of exposure to sexual traumatic events in particular among those who self-injure (Ellis et al., 2002; McReynolds & Wasserman, 2011; Morgan & Hawton, 2004).

We investigate the prevalence and correlates of NSSI among incarcerated male and female youth. In addition, we examine contributions of history of both physical and sexual violence, and of both internalizing and externalizing features, to NSSI. Further, as cutting is among the most prevalent method of NSSI in community youth (Barrocas, Hankin, Young & Abela, 2012), and likely to be the most prevalent among justice-involved youth (Matsumoto et al., 2005; McReynolds & Wasserman, 2011), we examine these associations among those who do and do not report lifetime nonsuicidal cutting.

## Method

### Context

Between May 2010 and December 2012, in a collaboration between the Illinois Department of Juvenile Justice (ILDJJ) and Columbia University, all new intakes into two secure juvenile facilities (one serving each gender) completed a diagnostic interview in the course of the standard assessment protocol that occurs upon arrival in a designated area within the facility. Consenting youth were further assessed on the Voiced Index of Self-injurious Actions (VISA: McReynolds & Wasserman, 2011), shortly after admission. Youth were not compensated for their participation. Further, youth were provided contact information for an individual who was not associated with the research study and who agreed to serve as a participant advocate should youth have any questions. The study was approved by our university’s Institutional Review Board.

### Participants

Altogether, 649 youth (281 females) were approached for VISA assessments. We excluded 22 youth with incomplete data on demographic and offense characteristics and 27 who reported that they had been less than “very truthful” in their VISA responses. Twelve additional individuals were excluded because their VISA responses indicated that their self-injury had occurred in the context of a suicide attempt. Of the remainder, information on disorder was available only for 385 youth. We report demographics, offense, NSSI characteristics, and diagnostic information for these 385 youth.

### Measures

#### Demographic and offense information

Local staff extracted information on gender, age, race, age at first offense, and repeat offender status from official justice records. Justice staff also provided information regarding “most serious” current offense, categorized as *interpersonal* (person- or weapon-related) versus all other types of offenses.

### Mental health status

Youth self-reported mental health status on the Voice Diagnostic Interview Schedule for Children (V-DISC). The DISC is a family of structured psychiatric interviews (e.g., Shaffer, Fisher, Lucas, Dulcan, & Schwab-Stone, 2000), based on the DSM-IV and has been widely used in juvenile justice settings (e.g., Teplin, Abram, McClelland, & Dulcan, 2002; Wasserman et al., 2010). With the V-DISC, which generates Axis-I disorders present in the past month, youth listen to pre-recorded questions over headphones as they appear on the computer screen and key in their responses. Twenty disorders in four diagnostic clusters were examined: Affective, Anxiety, Disruptive Behavior (DBD), and Substance Use Disorders (SUD), in addition to past-month and lifetime suicidal behavior.

We considered the following diagnostic indicators as potentially related to self-injury: SUD, Major Depressive Disorder (MDD), Conduct Disorder (CD), and Post-Traumatic Stress Disorder (PTSD). Suicide history items in the MDD module were excluded from that algorithm to avoid artificially inflating the relationship between MDD and suicidal behavior (Gould et al., 1998). As noted, each of these has been consistently empirically linked to adolescent NSSI.

### Traumatic exposure

V-DISC queries about lifetime history of eight traumatic exposures of which three consider exposures theoretically linked to NSSI. For exposure type, we considered two categorizations of assaultive violence, *Forced sexual activity* and *Nonsexual assault*. Youth reporting forced sex were considered exposed to forced sexual activity, while those who reported being attacked/beaten badly or being threatened by a weapon were considered exposed to nonsexual assault. Other types of traumatic exposure (e.g., experiencing a natural disaster, being in a bad accident or natural disaster, seeing someone get badly hurt, or seeing a dead body) were not considered because they have not been suggested as contributors to NSSI.

### Nonsuicidal self-injury

The Voiced Index of Self-injurious Actions (VISA: McReynolds & Wasserman, 2011) is an audio computer-assisted self-interview (ACASI) assessment, that youth self-administer by viewing questions on screen while also hearing them over headphones, and entering responses into the computer. The VISA inquires about the nature, frequency (lifetime and past-year), recency and age of onset of seven types of self-injury. Questions follow a format such as, “Have you ever… cut your skin with a sharp object on purpose?” Those reported self-injurious behavior in the past year are asked additional questions inquire about burning one’s self; eating or drinking something other than food, drugs, or medicine; biting one’s self, hitting one’s self, picking at skin, and putting something under the skin. For each behavior reported, the VISA inquires about the frequency of the behavior, injury and medical attention, and if the respondent had ever done this behavior when trying to kill him/herself. Self-injury types were aggregated into a single measure of NSSI. As noted above, we removed 12 individuals who reported (on the VISA) that their self-injury had been in the context of a suicide attempt.

Justice staff noted on logsheets whether or not, before taking the VISA, the youth was known to self-injure, as ascertained by case records, observation, or self-report to agency staff. Agency protocols also required that staff confirm, in a debriefing conversation, youth’s self-reported self-injurious behavior after VISA administration. Logsheets indicated that justice staff were aware, by one of these three means, of self-injury history for 86 youth (44 males, 42 females). The VISA assessment identified 17 additional individuals (9 males) with lifetime history of self-injury; of the total 103 identified through any means, then, 16.5% were newly identified by the VISA. The majority of those who reported self-injury on the VISA were confirmed by clinical staff: 80% of the males and 89% of the females who indicated lifetime history of one or another type of self-injury were confirmed, as were 93% and 95%, respectively, of males and females who reported having ever cut themselves on purpose (reflecting sensitivity between 80 and 95%).

### Statistical methods

Initial analyses examined overall sample characteristics and the two types of traumatic exposure differed by gender. Next, we examined whether sample characteristics differed among those who reported no self-injury, lifetime NSSI, and lifetime nonsuicidal cutting for the overall sample and within gender. Descriptive information about past-year acts of NSSI, and for cutting separately, are presented for the sample overall and by gender, and include age of onset, attempt to hide behavior, whether or not the act resulted in injury, and whether or not the injury required medical attention.

Multivariate regression models examined reported lifetime NSSI. In parallel analyses, we focused specifically on lifetime history of nonsuicidal cutting for two reasons. First, we anticipated that cutting would be the most frequent type of self-injury, and second, our measure of NSSI would otherwise include a mix of more and less serious forms of self-injury. From the pool of potential correlates (gender, age, race, age at first offense, repeat offender status, interpersonal current offense, psychiatric disorder, suicidal behavior, traumatic exposure) we selected those associated either theoretically or statistically (at *p* < .20) with either outcome in univariate regression analyses. Because of the strong overlap between gender and exposure to forced sexual activity (in a large national archive of justice-involved youth, females were ten times more likely to report forced sexual activity (Wasserman & McReynolds, 2011), both gender and traumatic exposure type could not be examined in the same analysis. We present overall, and gender-stratified, logistic regression models that include age, race (white vs non-white), MDD, lifetime suicide attempt, and exposure to forced sexual activity and nonsexual assault.

## Results

### Sample characteristics

Table 1 shows that the average participant was 17.3 years old (sd = 1.7), and more than 90% were repeat offenders. Two thirds were male, and nearly half were white (48%). For about half, the current most serious offense was interpersonal. Half reported a DSM-IV disorder, with approximately 20–28% endorsing CD or a SUD. Rates were expectably lower for MDD and PTSD (6.1% and 2.5%, respectively). Nine percent reported a lifetime suicide attempt and 1% reported a past-month suicide attempt on the V-DISC. Overall, approximately half reported exposure to nonsexual assault, and 15% reported exposure to forced sexual activity. Females were approximately seven times as likely to report exposure to forced sexual activity (χ^2^_(1)_ = 52.4, *p* < .001) and males were 1.5 times more likely to report exposure to nonsexual assault (χ^2^_(1)_ = 9.37, *p* < .01), although they did not differ significantly in exposure to assaultive violence in general (55.6% and 47.0% for males and females, respectively).

**Table 1:** Demographic, offense, clinical and traumatic exposure characteristics for overall sample and by NSSI status

	All participants (N = 358) n (%)	No self-injury (*N* = 266) n (%)	Lifetime NSSI (*n* = 92) n (%)	Lifetime nonsuicidal cutting^a^ (*n* = 49) n (%)
Female	116 (32.4)	79 (29.7)	37 (40.2)	(21) 42.9
Age (in years)^b^	17.2 (1.7)	17.3 (1.7)**	16.8 (1.8)	16.7 (1.9)
Highest academic grade^b^	9.7 (1.6)	9.7 (1.5)	9.6 (1.6)	9.7 (1.9)
White (vs nonwhite)	171 (47.8)	110 (41.4)	61 (66.3)***	41 (83.7)***
Repeat offender status	325 (91.8)	241 (91.3)	84 (93.3)	44 (89.8)
Age at first offense (in years) ^b^	13.0 (1.8)	12.9 (1.7)	13.1 (1.8)	13.1 (1.7)
Interpersonal offense	179 (50.1)	136 (51.3)	43 (46.7)	25 (51.0)
Mental health disorder or condition				
Any disorder	176 (49.2)	112 (42.1)	64 (69.6)***	30 (61.2)*
Post-traumatic Stress Disorder	9 (2.5)	7 (2.6)	2 (2.2)	1 (2.0)
Major Depressive Disorder*	22 (6.1)	10 (3.8)	12 (13.0)***	5 (6.1)
Conduct Disorder	73 (20.4)	47 (17.7)	26 (28.3)*	14 (28.6)
Substance Use Disorder	99 (27.7)	64 (24.2)	35 (38.0)*	18 (36.7)
Past 4 weeks suicide attempt	4 (1.1)	0 (0.0)	4 (4.3)***	1 (2.0)*
Lifetime suicide attempt	32 (9.0)	9 (3.4)	23 (25.0)***	14 (28.6)***
Traumatic Exposure				
Forced sexual activity	53 (14.8)	24 (9.1)	29 (31.5)***	16 (32.7)***
Nonsexual assault	174 (48.7)	117 (44.2)	57 (62.0)**	31 (63.3)*

*Note.* NSSI = Nonsuicidal self-injury. ^a^Individuals are a subset of those with lifetime NSSI and can have other nonsuicidal self-injurious behavior (i.e., they are not individuals who only engaged in nonsuicidal cutting); ^b^Mean (SD); ***p < 0.001; **p < 0.01; *p < 0.05 designate significant differences between NSSI group and non-injurers

### Rates of nonsuicidal self-injury

Table 2 shows that more than a quarter (25.7%) of the sample reported lifetime NSSI, with nonsuicidal cutting being the most frequent (13.7%). Among the 20 individuals reporting past-year nonsuicidal cutting, 15% did so on 6+ days, 30% made 6+ cuts on average when they cut, 55% tried to hide their cut(s), 57% bled, and 24% reported seeking medical attention for their cut(s). Rates of both lifetime NSSI and nonsuicidal cutting were somewhat (but not significantly) elevated among females (for NSSI: 31.9% for females vs. 22.7% for males; for nonsuicidal cutting: 18.1% for females vs. 11.6% for males).

**Table 2:** Characteristics of NSSI reported on the VISA

	Overall (*n* = 358)		Males (*n* = 242)		Females (*n* = 116)	
	*n* (%)		*n* (%)		*n* (%)	
	Any NSSI^a^	Nonsuicidal cutting	Any NSSI	Nonsuicidal cutting	Any NSSI	Nonsuicidal cutting
Lifetime	92 (25.7)	49 (13.7)	55 (22.7)	28 (11.6)	37 (31.9)	21 (18.1)
Past year	43 (12.0)	20 (5.6)	28 (11.6)	11 (6.0)	15 (12.9)	9 (7.8)
Age of onset (mean, sd)^b^	–	13.0 (2.1)	–	13.0 (2.0)	–	13.1 (2.4)
Nonsuicidal cutting on 6+ days in past year^b^	–	3 (15.0)	–	3 (27.3)	–	0 (0.0)
Made an average of 6+ cuts for past-year nonsuicidal cutting^b^	–	6 (30.0)	–	5 (45.5)	–	1 (11.1)
Tried to hide past-year NSSI	24 (38.5)	22 (54.5)	10 (27.8)	10 (45.5)	14 (45.2)	14 (63.6)
Past-year NSSI resulted in injury	38 (56.7)	28 (75.7)	17 (47.2)	13 (81.3)	21 (67.7)	15 (71.4)
Past-year NSSI required medical attention	18 (26.9)	9 (24.3)	5 (16.1)	2 (12.5)	13 (41.9)	7 (33.3)

*Note*. *NSSI* nonsuicidal self-injury, *VISA v*oiced index of self-injurious actions. ^a^Any NSSI includes cutting, burning, ingesting non-food substances, biting, self-hitting, pick at one’s skin, and placed objects under one’s skin; ^b^Characteristics specifically refer to nonsuicidal cutting

### Comparing those who do and do not perform nonsuicidal self-injury

Table 1 also presents information on whether demographic, offense, and disorder characteristics differed among those who reported no NSSI, lifetime NSSI, and lifetime non-suicidal cutting for the overall sample. Compared to those who did not self-injure, those reporting lifetime NSSI were more likely to be younger (*t*_(356)_ = 2.74, *p* < .01), white (*χ* ^2^_(1)_ = 17.06, *p* < .001), endorse a DSM-IV disorder (*χ* ^2^_(1)_ = 20.62, *p* < .001), MDD (*χ* ^2^_(1)_ = 10.22, *p* < .001), CD (*χ* ^2^_(1)_ = 4.65, *p* < .05), SUD (*χ* ^2^_(1)_ = 6.58, *p* < .01), a lifetime suicide attempt (*χ* ^2^_(1)_ = 39.06, *p* < .001), a recent suicide attempt (*χ* ^2^_(1)_ = 11.65, *p* = .001), and report both forced sexual activity (31.5% vs 9.1%, χ^2^_(1)_ = 27.26, *p* < .001) and nonsexual assault (62.0% vs 44.2%, χ^2^_(1)_ = 8.67, *p* < .01). The group differences remained for comparisons between those who reported nonsuicidal cutting and non-injurers, except that differences in age, and elevated rates of MDD, CD, and SUD were no longer statistically significant (bivariate statistics for these comparisons available upon request).

NSSI group comparisons of sample characteristics stratified by gender (Table 3) showed some gender-neutral and gender-specific risks for NSSI. For both males and females, compared to non-injurers, those reporting lifetime NSSI were more likely to be white (χ^2^_(1)_ = 9.69, *p* < .01 and χ^2^_(1)_ = 10.70, *p* < .001 for males and females respectively), endorse a DSM-IV disorder (χ^2^_(1)_ = 13.30, *p* < .001 and χ^2^_(1)_ = 6.81, p < .01 for males and females respectively), SUD (χ^2^_(1)_ = 3.90, *p* < .05 and χ^2^_(1)_ = 4.02, p < .05 for males and females respectively), a lifetime suicide attempt (χ^2^_(1)_ = 21.38, p < .001 and χ^2^_(1)_ = 15.73, p < .001 for males and females respectively), and forced sexual activity (χ^2^_(1)_ = 4.25, p < .05 and χ^2^_(1)_ = 18.42, p < .001 for males and females respectively). Male-specific risks for lifetime NSSI included age (*t*_(240)_ = 2.80, *p* < .01), MDD (χ^2^_(1)_ = 7.58, p < .01), CD (χ^2^_(1)_ = 5.18, *p* < .05), a recent suicide attempt (χ^2^_(1)_ = 10.33, p < .001), and nonsexual assault (χ^2^_(1)_ = 9.69, *p* < .01).

**Table 3:** Demographic, offense, clinical and traumatic exposure characteristics by NSSI status within gender

	Males			Females		
	No self-injury (*n* = 266) *n* (%)	Lifetime NSSI (*n* = 55) *n* (%)	Lifetime nonsuicidal cutting^a^ (*n* = 37) *n* (%)	No self-injury (*n* = 266) *n* (%)	Lifetime NSSI (*n* = 37) *n* (%)	Lifetime nonsuicidal cutting^a^ (*n* = 21) *n* (%)
Age (in years)^b^	17.6 (1.6)**	16.9 (1.9)	16.8 (2.0)	16.6 (1.5)	16.6 (1.6)	16.7 (1.9)
Highest academic grade^b^	10.0 (1.5)	9.9 (1.6)	9.6 (1.9)	9.2 (1.6)	9.5 (1.6)	9.7 (1.8)
White (vs nonwhite)	88 (47.1)	39 (70.9)**	26 (92.9)***	22 (27.8)	22 (59.5)***	15 (71.4)***
Repeat offender status	178 (95.2)	53 (96.4)	26 (92.9)	63 (81.8)	31 (88.6)	18 (85.7)
Age at first offense (in years)^b^	12.8 (1.8)	12.8 (1.9)	12.8 (1.9)	13.1 (1.8)	13.5 (1.4)	13.5 (1.3)
Interpersonal offense	95 (51.1)	25 (45.5)	14 (50.0)	41 (51.9)	18 (48.6)	11 (52.4)
Mental health disorder or condition						
Any disorder	77 (41.2)	38 (69.1)***	17 (60.7)	35 (44.3)	26 (70.3)**	13 (51.9)
Post-traumatic Stress Disorder	3 (1.6)	2 (3.6)	1 (3.5)	4 (5.1)	0 (0.0)	0 (0.0)
Major Depressive Disorder	6 (3.2)	7 (12.7)**	3 (7.1)	4 (5.1)	5 (13.5)	2 (4.8)
Conduct Disorder	29 (15.5)	16 (29.1)*	8 (28.6)	18 (23.1)	10 (27.0)	6 (28.6)
Substance Use Disorder	52 (27.8)	23 (41.8)	12 (42.9)	12 (15.4)	12 (32.4)*	6 (28.6)
Past 4 weeks suicide attempt	0 (0.0)	3 (5.5)**	1 (3.6)**	0 (0.0)	1 (2.7)	0 (0.0)
Lifetime suicide attempt	6 (3.2)	12 (21.8)***	8 (28.6)***	3 (3.8)	11 (29.7)***	6 (28.6)***
Traumatic Exposure						
Forced sexual activity	7 (3.8)	6 (10.9)*	3 (10.7)	17 (21.5)	23 (62.2)***	13 (61.9)***
Nonsexual assault	91 (48.9)	40 (72.7)**	20 (71.4)*	26 (32.9)	17 (45.9)	11 (52.4)

*Note. NSSI* nonsuicidal self-injury. ^a^ Individuals are a subset of those with lifetime NSSI and can have other nonsuicidal self-injurious behavior (i.e., they are not individuals who only engaged in nonsuicidal cutting); ^b^Mean (SD); ***p < 0.001; **p < 0.01; *p < 0.05 designate significant differences between NSSI group and non-injurers within gender

Two of the gender-neutral risks remained for comparisons between those reporting lifetime nonsuicidal cutting and non-injurers (Table 3). Those reporting nonsuicidal cutting were more likely to be white (χ^2^_(1)_ = 20.51, *p* < .001 and χ^2^_(1)_ = 13.52, p < .001 for males and females respectively) and report a lifetime suicide attempt (χ^2^_(1)_ = 25.73, *p* < .001 and χ^2^_(1)_ = 12.24, *p* < .001 for males and females respectively). For nonsuicidal cutting, there were three instances of gender-specific risks. Males reporting nonsuicidal cutting were younger (*t*_(213)_ = 2.64, *p* < .01), and more likely to report a recent suicide attempt (χ^2^_(1)_ = 6.71, *p* < .01) and nonsexual assault (χ^2^_(1)_ = 4.94, *p* < .05) compared to non-injuring males. Females reporting nonsuicidal cutting reported a higher rate of exposure to forced sexual activity (χ^2^_(1)_ = 12.89, *p* < .001) compared to non-injuring females.

### Multivariate analyses

Table 4 presents final models for logistic regressions comparing first those reporting lifetime NSSI, and then those reporting lifetime nonsuicidal cutting, to non-injurers for the overall sample and then stratified by gender. Those with a history of NSSI were a few months younger, almost three times as likely to be white, more than seven times as likely to have made a lifetime suicide attempt, and approximately twice as likely to report exposure to either forced sexual activity or nonsexual assault. Results were similar for nonsuicidal cutting, except that neither type of traumatic exposure (nonsexual assault or forced sexual activity) was significantly associated with NSSI, likely a consequence of lower power. These features explained approximately 28% of the variance in NSSI and 36% of the variance in nonsuicidal cutting.

**Table 4:** Multivariate regressions predicting nonsuicidal self-injury (NSSI) for overall sample and stratified by gender

	Predicting lifetime NSSI (vs no self-injury)					
	Overall (*n* = 356)		Males (*n* = 241)		Females (*n* = 115)	
Nagelkerke R^2^ (%)	27.6		27.4		35.4	
	OR	95% CI	OR	95% CI	OR	95% CI
Age (in years)	0.81*	[0.69, 0.96]	0.74**	[0.60, 0.90]	1.03	[0.75, 1.40]
White (vs non-white)	2.77***	[1.59, 4.82]	3.02**	[1.42, 6.43]	3.44*	[1.34, 8.80]
Major Depressive Disorder	2.60	[0.92, 7.39]	4.29*	[1.16, 15.88]	0.98	[0.16, 6.08]
Lifetime suicide attempt	7.17***	[2.95, 17.40]	7.02***	[2.22, 22.23]	7.36*	[1.60, 33.77]
Traumatic exposure						
Forced sexual activity	2.37*	[1.18, 4.76]	1.32	[0.37, 4.73]	4.89*	[1.44, 16.55]
Nonsexual assault	1.85*	[1.05, 3.24]	2.73**	[1.31, 5.69]	0.70	[0.21, 2.34]
	Predicting lifetime nonsuicidal cutting (vs no self-injury)					
	Overall (*n* = 313)		Males (*n* = 214)		Females (*n* = 99)	
Nagelkerke R^2^ (%)	36.0		41.1		40.3	
Age (in years)	0.78*	[0.62, 0.97]	0.67**	[0.51, 0.89]	1.00	[0.68, 1.46]
White (vs non-white)	8.03***	[3.30, 19.54]	22.82***	[3.90, 133.39]	7.76**	[2.19, 27.45]
Major Depressive Disorder	2.39	[0.56, 10.27]	12.21*	[1.52, 97.74]	0.59	[0.07, 5.32]
Lifetime suicide attempt	9.77***	[3.46, 27.63]	10.99***	[2.90, 41.61]	10.81*	[1.69, 69.01]
Traumatic exposure						
Forced sexual activity	2.34	[0.93, 5.90]	0.59	[0.09, 3.86]	3.96	[0.87, 18.00]
Nonsexual assault	1.85	[0.86, 3.98]	2.78	[0.98, 7.87]	1.18	[0.28, 5.06]

*Note*. *OR* odds ratio, *CI* confidence interval; *p < .05. **p < .01. ***p < .001

Comparison of gender-stratified regression analyses showed that those with a history of NSSI were approximately three times as likely to be white and more than seven times as likely to have made a lifetime suicide attempt. Three correlates remained significant risks for males only; males who reported lifetime NSSI were slightly younger, over four times as likely to report MDD, and nearly three times more likely to report nonsexual assault. For females only, those reporting lifetime NSSI were nearly five times more likely to report exposure to forced sexual activity. Similar to overall analyses for nonsuicidal cutting, neither type of traumatic exposure (nonsexual assault or forced sexual activity) was significantly associated with nonsuicidal cutting for both genders. These features explained 27% and 35% of the variance in lifetime NSSI for males and females respectively, and 41% and 40% of the variance in nonsuicidal cutting for males and females respectively.

## Discussion

### Rates of NSSI in incarcerated juveniles

The rates of lifetime NSSI reported here (12% and 26% respectively) appear at higher end of the range that has been reported for community counterparts. In community adolescent samples, rates of lifetime NSSI range between 13 and 32% for females and between 13 and 23% for males (e.g., Jacobson & Gould, 2007; Sornberger et al., 2012). Swannell et al. (2014) report a pooled prevalence of 17.2%. The present results fall within the range reported in a recent review (Casiano et al., 2013) of “deliberate self-harm” (acts of both suicidal and nonsuicidal self-injury) in incarcerated juveniles. Most investigations in that review reported rates between 10% and 40%. This wide range is likely a consequence of methodological differences, as has been noted in reviews of both community adolescent (Muehlenkamp, Claes, Havertape, & Plener, 2012) and adult forensic (Dixon-Gordon, 2011) studies. Methodological factors (e.g., inquiry format, sample characteristics, and lack of distinction between persons whose self-harming behavior is suicidal and those for whom it is not suicidal in intent) can account for more than 50% of the heterogeneity in prevalence estimates (Swannell et al., 2014).

The present report observes similar rates of NSSI for both genders. It is likely that gender differences in other reports reflect differences in comorbid conditions, such as internalizing disorder and suicidal behavior, not adjusted for in comparisons. Among justice-involved youth, rates of internalizing disorder and suicidal behavior are commonly reported to be higher among females (Teplin et al., 2002; Vincent, Grisso, Terry, & Banks, 2008; Wasserman et al., 2010), and in the current sample, females reported higher rates of both internalizing disorder (29.3% vs 19.4%) and lifetime suicide attempt (12.2% vs 7.4%). In recognition of this comorbidity, we adjusted for the role of MDD and suicidal behavior when considering associations with NSSI. Other studies’ lack of adjustment for these comorbid conditions in multivariate analyses might have resulted in observed elevated rates of NSSI in females.

### Associations with mood disorder and suicidal behavior

In non-forensic samples of adolescents, depressive symptoms are often identified as a significant risk for self-injury (Brunner et al., 2007; Garrison et al., 1993; Hilt, Cha, & Nolen-Hoeksema, 2008; Ross & Heath, 2003). Here, among incarcerated youth, those who reported NSSI (or nonsuicidal cutting) were more than twice as likely to meet criteria for MDD, and more than seven times as likely to report a suicide attempt history, compared to non-injurers. Despite excluding individuals who reported that their self-injury had taken place in the context of a suicide attempt, the NSSI group nonetheless retained 23 individuals who had reported a lifetime suicide attempt on the V-DISC. These individuals may have employed means for suicide attempts not referenced on the VISA (e.g., jumping, hanging, shooting) for their earlier suicide attempts, or they may also have engaged in both types of behavior at different times: they have attempted suicide, but they had at least one instance when they self-injured while not intending to die. Moreover, gender-specific multivariate analyses highlight the particular risk for NSSI associated with having a MDD among males.

### Associations with traumatic exposure

Rates of exposure (and gender differences in those rates) to assaultive violence are comparable to those reported in a large national multi-site juvenile justice sample (Wasserman & McReynolds, 2011). Here, although gender differences did not appear for exposure to assaultive violence in general, females were far more likely to report exposure to forced sexual activity, while males were more likely to report exposure to nonsexual assault. A widely-cited model of the etiology of self-injury highlights the role of sex abuse (van der Kolk et al., 1991). Among community adolescents, abuse (and particularly sexual abuse) history is sometimes found to be more common among those who engage in self-injury in general (Asgeirsdottir et al., 2011) as well as among those engaging in NSSI (Zoroglu et al., 2003). In juvenile forensic samples, history of both sexual and physical abuse are more common among those reporting self-injury in general (Morgan & Hawton, 2004); and a history of sexual abuse is more strongly associated with NSSI for females than for males (Ellis et al., 2002). In our earlier work with similar methods and analytic strategy, both physical and sexual abuse history were significantly more likely among incarcerated female adolescents (that study did not include males) who reported some form of self-injury on the VISA (McReynolds & Wasserman, 2011). In the current sample, when both sexual and nonsexual assault history were added to predictive models, each was independently associated with NSSI. There are few investigations of NSSI that have considered a measure of both physical and sexual abuse. Similar to a study among incarcerated female adolescents (McReynolds & Wasserman, 2011), we found that those who cut were significantly more likely to report both sexual and physical abuse. Here, we extend knowledge of gender-specific risk for NSSI to include exposure to nonsexual assault for males.

### Consistency with DSM-5 proposed criteria

Among those with a history of past-year NSSI, nearly a quarter indicated that they had done so on six or more days. DSM-5 proposed criteria (American Psychiatric Association, 2013) restrict this designation to those who had done so on five or more days, so that the present sample may include some who would not meet the proposed criteria, because of less frequent self-injury. Consistent with DSM-5 criteria, here most (85%) persons reporting past-year nonsuicidal self-injury did not seek medical attention. While the DSM-5 posits that gender differences are less marked for NSSI than for suicidal behavior, we find similar gender disparities for both types of behavior. Here, in unadjusted analyses, females were approximately 28% more likely to report lifetime history of NSSI, 33% more likely to report lifetime nonsuicidal cutting, and were 36% more likely to report a lifetime suicide attempt. While not significant, both suicidal and nonsuicidal self-injury are slightly elevated among females. A recent review (Swannell et al., 2014) suggests that, while clinical samples of those engaging in NSSI include a preponderance of females, gender equivalence is more characteristic of samples not selected for presence of disorder, such as the one studied here. Overall then, although this sample may include individuals whose self-injury is less frequent than proposed in the DSM-5, most features are consistent with that depiction.

### Limitations

First, the high degree of collinearity between traumatic exposure and gender prohibited examination of gender-specific interactions via multivariate regression. This limitation was further compounded by the relatively fewer number of females in our sample, a condition common to studies of youth in the juvenile justice system; future work with larger samples might address this limitation. Next, the DSM-5’s proposed criteria for NSSI require that the behavior occur on five or more days in the past year, although many in the present sample did not report doing so. Only 10 of those designated as showing NSSI reported behavior at this level of persistence, so that the present sample likely includes individuals whose NSSI is less severe than those who would meet proposed DSM-5 criteria. Finally, there is the possibility that rates of NSSI are underreported due to the time lag between behavior and VISA administration, however this recall bias would most likely serve to diminish observed associations.

### Implications for clinical policy

Over the past 20 years, as research has accumulated on the high prevalence of disorder and suicide risk among youth in contact with the juvenile justice system, agencies have increasingly moved to standardize their services related to the identification and treatment support for mental health needs for youth in their care (e.g., Skowyra & Cocozza, 2006). The present results point to the need for refinement of clinical policy in two related areas. First, although we report only a slightly higher prevalence of NSSI than found in community samples, instances of self-injury that appear in group living situations can be very disruptive for both staff and residents, and, like suicide attempts, may prompt social contagion. Exposure to media reports (Gould, Jamieson, & Romer, 2003) or other means of social communication (Hasking, Andrews, & Martin, 2013) have been found to prompt self-harming behaviors in others (see Jarvi, Jackson, Swenson, & Crawford, 2013, for a review). This means that justice agencies should have protocols in place that support both systematic identification of NSSI history as well as its management. Moreover, given the variability in ascertainment of NSSI that can be attributed to small differences in assessment methods, agencies should set in place identification protocols that rely on standardized assessment procedures. Agencies would be better served by using a checklist or a standard set of questions, rather than leaving the means for ascertainment up to the individual clinician or intake worker. Second, we found only modestly elevated rates of NSSI among females, reflecting the increased risk for both disorder and traumatic exposure among incarcerated juveniles of both genders. As a consequence, juvenile agencies should apply their assessment protocols to *both* females and males.

## Conclusion

This paper helps better characterize this new DSM-5 diagnostic area of NSSI, supports the lack of gender-differences among incarcerated youth, and considers important correlates such as traumatic exposure. This work suggests the importance of including screening both genders for NSSI as part of a comprehensive, scientifically-sound behavioral health screen for justice-involved youth.
